# Does the grass snake (*Natrix natrix*) (Squamata: Serpentes: Natricinae) fit the amniotes-specific model of myogenesis?

**DOI:** 10.1007/s00709-016-1040-5

**Published:** 2016-11-10

**Authors:** Damian Lewandowski, Magda Dubińska-Magiera, Ewelina Posyniak, Weronika Rupik, Małgorzata Daczewska

**Affiliations:** 1 Department of Animal Developmental Biology, Institute of Experimental Biology, University of Wroclaw, Sienkiewicza 21, 50-335 Wroclaw, Poland; 2 Department of Animal Histology and Embryology, University of Silesia, Bankowa 9, 40-007 Katowice, Poland

**Keywords:** Amniotes, Myotomal myogenesis, Pax3/7 proteins, Satellite cells, Reptiles, Snake

## Abstract

In the grass snake (*Natrix natrix*), the newly developed somites form vesicles that are located on both sides of the neural tube. The walls of the vesicles are composed of tightly connected epithelial cells surrounding the cavity (the somitocoel). Also, in the newly formed somites, the Pax3 protein can be observed in the somite wall cells. Subsequently, the somite splits into three compartments: the sclerotome, dermomyotome (with the dorsomedial [DM] and the ventrolateral [VL] lips) and the myotome. At this stage, the Pax3 protein is detected in both the DM and VL lips of the dermomyotome and in the mononucleated cells of the myotome, whereas the Pax7 protein is observed in the medial part of the dermomyotome and in some of the mononucleated cells of the myotome. The mononucleated cells then become elongated and form myotubes. As myogenesis proceeds, the myotome is filled with multinucleated myotubes accompanied by mononucleated, Pax7-positive cells (satellite cells) that are involved in muscle growth. The Pax3-positive progenitor muscle cells are no longer observed. Moreover, we have observed unique features in the differentiation of the muscles in these snakes. Specifically, our studies have revealed the presence of two classes of muscles in the myotomes. The first class is characterised by fast muscle fibres, with myofibrils equally distributed throughout the sarcoplasm. In the second class, composed of slow muscle fibres, the sarcoplasm is filled with lipid droplets. We assume that their storage could play a crucial role during hibernation in the adult snakes. We suggest that the model of myotomal myogenesis in reptiles, birds and mammals shows the same morphological and molecular character. We therefore believe that the grass snake, in spite of the unique features of its myogenesis, fits into the amniotes-specific model of trunk muscle development.

**Electronic supplementary material:**

The online version of this article (doi:10.1007/s00709-016-1040-5) contains supplementary material, which is available to authorized users.

## Introduction

Reptiles are a very heterogeneous group of vertebrates (Olmo 2008). They were the first vertebrates to settle in the terrestrial environment. This shaped their musculoskeletal, respiratory and circulatory systems and their method of reproduction in a manner independent of the conditions of an aquatic environment. However, reptiles are still ectothermic organisms, which are strongly dependent on the environmental temperature. From an evolutionarily viewpoint, reptiles were the first terrestrial vertebrates to display a great diversity of muscle models, which correspond to their various modes of locomotion (crocodiles, turtles, lizards and snakes) (McNeill 2012). This great anatomical-physiological diversity of the muscular system renders it difficult to select a characteristic species that is representative of the whole taxon (Schilling 2011). As a result, the differentiations of the reptilian skeletal and limb muscles are still poorly understood, even though reptiles are crucial organisms in vertebrate evolution.

The muscle fibres of vertebrates are cylindrical, multinucleated cells that are divided into two main types: red (slow) and white (fast) muscle fibres (Yablonka-Reuveni 2011). White muscle fibres are characterised by a fast contraction, an anaerobic metabolism for energy, lower amounts of myoglobin and fewer mitochondria. They perform fast and intensive work, but only for a short period of time; therefore, they quickly experience fatigue. In contrast, the red muscle fibres are rich in mitochondria and myoglobin. They are dependent on an aerobic metabolism and can perform slow and sustained contractions for a prolonged period of time without fatigue (Sänger and Stoiber 2001). The proportion of white to red muscles in a vertebrate organism depends on its lifestyle. In fish, the following phenomena are frequently observed: in the eel, metamorphosis is accompanied by an increase in the amount of red muscles, as preparation for the spawning migration (Lewander et al. 1974); in the tuna, which is the most active species of fish, an extra band of red muscles can be observed near to the vertebral column (as reviewed by Katz 2002); and in salmonids, as migrant fish, extra red muscles are found as individual fibres distributed throughout the white muscles (Webb 1971).

It has been observed in lizards that, as terrestrial animals, the axial muscles stabilise the trunk during locomotion (Ritter 1996). A body of evidence has revealed that lizards possess both slow and fast muscles, whereas in snakes, white muscle fibres represent the prominent group of muscles in the myotomes (Gleeson et al. 1980; Guthe 1981; Moritz and Schilling 2013). Crow and Stockdale (1986) revealed that, in avians, there is no single program of fast myosin heavy chains (MyHC) isoform expression during development. Furthermore, recent studies on mammals have revealed significant species-dependent diversity in the distribution of muscle fibres (Schiaffino and Reggiani 2011).

In all vertebrates, the myotomal muscles have a similar structure and originate from the paraxial mesoderm, which undergoes segmentation into repetitive units called somites (Mok and Sweetman 2011; Bentzinger et al. 2012). However, the mechanism of somite differentiation is different in non-amniotes than in amniotes. For instance, the muscle fibre precursors of *Xenopus laevis* and *Danio rerio* undergo a 90° rotation (Bryson-Richardson and Currie 2010). The other characteristic feature of non-amniote myogenesis is the trans-myotomal migration of the red muscle precursors to the peripheral part of the myotome. In amniotes, as in the non-amniotes, the somites differentiate into the following: the epithelial dermomyotome (the source of skin connective tissues and the myotomal muscles) with characteristic dorsomedial (DM) and ventrolateral (VL) lips and the mesenchymal sclerotome (the source of the connective tissues of the axial skeleton) (Cinnamon et al. 2001; Ordahl et al. 2001; Steinbacher et al. 2006; 2007). The main part of the somite is occupied by the myotome, where the myotomal (trunk) muscles are differentiated in situ. It has been clearly established that in vertebrates, the dermomyotome is an ancient, conserved structure (Onai et al. 2015). Also, many different lines of evidence (from investigations on fish, amphibian, birds and mammals) have shown that the dermomyotome is the main source of the muscle progenitor cells (Gros et al. 2005; Relaix et al. 2005; Kassar-Ducchossoy et al. 2005; Kalcheim and Ben-Yair 2005; Manceau et al. 2008; Rossi and Messina 2014). The muscle progenitor cells express the transcription factors Pax3 and Pax7 (Tajbakhsh et al. 1997; Pownall et al. 2003; Halevy et al. 2004; Buckingham and Relaix 2007). The muscles then grow through the fusion of the muscle precursors with the existing muscle fibres (hypertrophy), resulting in an increase in the number of nuclei in the growing muscles. Additionally, during hyperplasia, the muscle precursors fuse with each other to form new muscle fibres (Greer-Walker 1970; Koumans et al. 1993; Stickland 1983).

Although a number of good studies on the anatomic description of somites and myotome in reptiles have been published, little work has been done in this class of animals that examines in parallel the immune-histological characterisation of those structures (Eckalbar et al. 2012; Rupik et al. 2012). Therefore, the key aim of our studies was to fill the gap in knowledge on muscle differentiation in this taxon by investigating muscle growth and its differentiation in the grass snake (*Natrix natrix*).

## Materials and methods

### Study animals

Fertilised females of the grass snake, *N. natrix* Stejneger, 1907, were caught in Poland in the vicinity of Wrocław at the beginning of May 2014. All of the specimens used in the experiments were captured according to the Polish regulations concerning the protection of wild species (Journal of Laws 1991, No. 114 Item 492; Journal of Laws 2000, No. 66 Item 802; Journal of Laws 2004, No. 112 Item 1183; and Journal of Laws 2015, No. 133 Item 266). The Department of Animal Developmental Biology of the University of Wroclaw obtained approvals from the Local Ethics Commission in Wroclaw (77/2013) and the Polish Ministry of Environment to perform studies on a protected species (Ref. No: WPN.6401.59.2014.IW and DZP-WG.6401.02.3.2014.JRO). The animals were kept in vivaria in an open farm area, in conditions similar to those in the wild until the eggs were laid, and then, they were released into their native area. The eggs of the grass snakes (*n* = 128) after oviposition were carefully collected and were placed inside plastic boxes filled with moistened perlite (at 100% humidity) with ventilation holes. The eggs were incubated at 30 °C, reflecting the seasonal ambient temperatures in the wild. The embryos used for the examination were isolated at regular intervals after the egg laying, until the 14th day after oviposition (stages I–VI). The developmental stages of the embryos were estimated using the developmental table published by Rupik (2002).

The collected embryos were anaesthetised with tricaine methanesulfonate (MS-222; 500 μg per gram of body weight) (Conroy et al. 2009), before being decapitated and dissected for further analysis (Journal of Laws 2015, No. 133 Item 266).

### Light and transmission electron microscopy

For light and electron microscopic examination, small pieces of the embryonic body wall, including differentiated muscle tissues, were fixed in a modified Karnovsky fixative consisting of 1% paraformaldehyde (PFA) and 1% glutaraldehyde, in a 0.1 M phosphate buffer (pH 7.2) for 24 h at 4 °C. The material was then repeatedly rinsed with the same buffer and was postfixed for 2 h in a 1:1 mixture of osmium tetroxide-potassium ferricyanide [*OsO4*-K3Fe(CN)6]. Following rinsing in the phosphate buffer, the material was dehydrated, first in a graded alcohol series and then in acetone, and was then embedded in epoxy resin Epon 812 (Sigma-Aldrich) (Luft 1961). The Epon blocks were cut on a Leica Ultracut UCT (Leica, Wetzlar, Germany). Semi-thin sections (0.6 μm) were collected on glass slides and were stained with methylene blue in a 1% borax solution before being examined under an Olympus BX60 light microscope (Olympus). Additionally, ultra-thin sections were collected on 200-mesh copper grids and were stained with uranyl acetate and lead citrate according to the standard protocol (Reynolds 1963), before being examined under a transmission electron microscope, Zeiss EM 900 (Carl Zeiss AG, Oberkochen, Germany; 80 kV).

### Immunofluorescence analysis

After the dissection and fixation of the embryos in 4% PFA in phosphate buffered saline (PBS) for 45 min at room temperature, the samples were transferred to 30% sucrose in PBS (for an overnight incubation at 4 °C). Next, samples were embedded in the optimal cutting temperature (OCT) medium and were placed in a cryomold and frozen. The samples were cut into 10-μm sections in a cryostat at −20 °C and were placed on SuperFrost Plus slides and subjected to immunofluorescence staining.

Standard immunofluorescence reactions were carried out on whole embryos or on tissue cryosections. These samples were blocked with 1% bovine serum albumin (BSA) in PBST (PBS with 0.1% Tween-20) for 60 min at room temperature. All of the wash steps were done with PBST. An incubation with primary antibodies was conducted overnight at 4 °C and with secondary antibodies for 60 min at room temperature. The following primary antibodies were used: mouse monoclonal anti-Pax3 antibody (Developmental Studies Hybridoma Bank) at a dilution of 1:50 in PBST, rabbit polyclonal anti-phospho-histone H3 [pSer10] (Sigma-Aldrich) at a dilution of 1:200 in PBST, mouse monoclonal anti-fast skeletal myosin (Fast MyHC) antibody (Abcam) at a dilution of 1:100 in PBST, mouse monoclonal anti-slow skeletal myosin (Slow MyHC) antibody (Abcam) at a dilution of 1:100 in PBST and mouse monoclonal F59 antibody (Developmental Studies Hybridoma Bank) at a dilution of 1:100 in PBST. Additionally, the following secondary antibodies were used: goat anti-mouse IgG FITC conjugated (Sigma-Aldrich) at a dilution of 1:50 in PBST, goat anti-rabbit IgG TRITC conjugated (Sigma-Aldrich) at a dilution of 1:50 in PBST and donkey anti-mouse IgG Cy5 conjugated (Jackson ImmunoResearch) at a dilution of 1:100 in PBST. For the F-actin identification, Alexa Fluor 546-conjugated phalloidin and Alexa Fluor 488-conjugated phalloidin were used (Molecular Probes) at a dilution of 1:80 in PBS. The DNA was stained with 4,6-diamidino-2-phenylindole (DAPI; 0.2 μg/ml), and the neutral lipids were stained with BODIPY® 493/503 (Thermo Fisher Scientific) at a dilution 1:1000. The samples were mounted in a fluorescent mounting medium (Dako). For the imaging, an Olympus FluoView FV1000 confocal laser scanning microscope (Olympus) was used. The images were recorded by employing the Plan-Apochromat ×10, ×20 or ×40 objectives. Any brightness and contrast adjustments were performed in the FV10-ASW_Viewer or in ImageJ.

### Gel electrophoresis and Western blotting

The decapitated embryo extracts underwent a Western blot analysis. The proteins were separated on 12% SDS gels by polyacrylamide gel electrophoresis (PAGE) and were electrotransferred onto nitrocellulose filters. The membranes were then detected and documented with a chemiluminescent method, using the Bio-Rad ChemiDoc Imaging System. The protein content in the Pax3 and Pax7 bands was then normalised according to the α-actinin content in each lane.

The membranes were blocked for 60 min at room temperature in a blocking solution (5% non-fat dry milk in PBS with 0.05% Tween-20). The membranes were then incubated overnight at 4 °C with primary monoclonal antibodies specific for Pax3 (mouse monoclonal anti-Pax3 antibody [Developmental Studies Hybridoma Bank] at a dilution of 1:100), Pax7 (mouse monoclonal anti-Pax7 antibody [Developmental Studies Hybridoma Bank] at a dilution of 1:100) and α-actinin (rat monoclonal anti-α-actinin antibody [Babraham Bioscience Technologies] at a dilution of 1:100). Chemiluminescence was then used for the detection of Pax3, Pax7 and α-actinin, by the incubation of the membrane for 60 min at room temperature with the following secondary antibodies: donkey anti-mouse IgG peroxidase (HRP) conjugated (Jackson ImmunoResearch) and donkey anti-rat IgG peroxidase (HRP) conjugated (Jackson ImmunoResearch) at a dilution of 1:10,000.

## Results

### Somitogenesis and myogenesis

In the studied species (*N. natrix*), selected stages (from stage I to VII) of the skeletal muscle development were analysed using the light, confocal and transmission electron microscope and with the Western blot method.

**Fig. 1:**
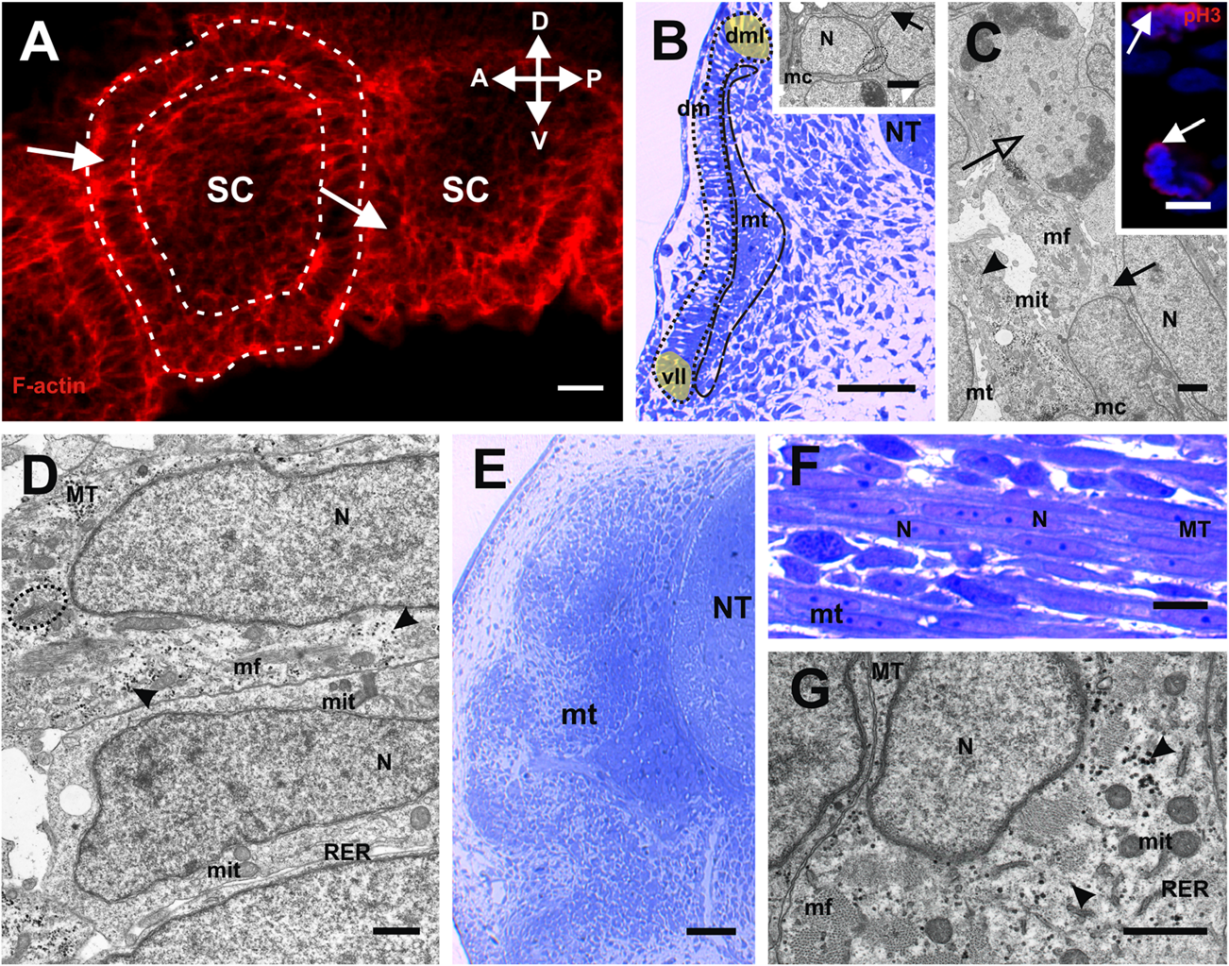
Somitogenesis and myogenesis. **a** Stage I. Structure of posterior somites (*white arrows*). Somites form vesicles (surrounded with *dashed line*) with centrally located somitocoel (*SC*). F-actin (*red*), anterior (*A*), posterior (*P*), dorsal (*D*), ventral (*V*). Whole mount staining, confocal microscope. *Scale bar*: 20 μm. **b** Stage II. Structure of anterior part of embryo. Laterally located dermomyotome (*dm*; surrounded with *dotted line*) with ventrolateral (*vll*; highlighted in *light yellow*) and dorsomedial (*dml*; highlighted in *light yellow*) lips. *NT* neural tube, *mt* myotome (surrounded with *dashed line*). Transverse, semithin section, methylene blue staining. *Scale bar*: 50 μm. *Inset*: ultrastructure of myotome. Nucleus (*N*), rough endoplasmic reticulum (*black arrowhead*), mononucleated cells (*mc*), Golgi apparatus (*encircled*). Transverse, ultrathin section, TEM. *Scale bar*: 2 μm. **c** Stage III. Ultrastructure of posterior myotome (*mt*) with numerous organelles in mononucleated cells (*mc*) cytoplasm. Nucleus (*N*), rough endoplasmic reticulum (*black arrow*), mitochondria (*mit*), myofibrils (*mf*), glycogen granules (*black arrowhead*), divided cell in telophase stage (*empty arrow*). Longitudinal, ultrastructure section, TEM. *Scale bar*: 2 μm. *Inset*: immunodetection of phosphorylated histone H3 (*red*) in the posterior myotome. Nuclei (*blue*). Transverse, cryosection, confocal microscope. *Scale bar*: 5 μm. **d** Stage III. Ultrastructure of posterior myotome filled with mononucleated myotubes (*MT*) with elongated nuclei (*N*). Numerous organelles visible in the cytoplasm: mitochondria (*mit*), Golgi apparatus (*encircled*), rough endoplasmic reticulum (*RER*), glycogen granules (*black arrowheads*), myofibrils (mf). Longitudinal, ultrathin section, TEM. *Scale bar*: 1 μm. **e** Stage IV. Structure of posterior part of embryo. Neural tube (*NT*), myotome (*mt*). Transverse, semithin section, methylene blue staining. *Scale bar*: 50 μm. **f** Stage V. Structure of anterior part of embryo. Myotome (*mt*) is filled with multinucleated myotubes (*MT*). Nuclei (*N*). Longitudinal semithin section, methylene blue staining. *Scale bar*: 10 μm. **g** Stage V. Ultrastructure of posterior myotome. Sarcoplasm of multinucleated myotube (*MT*) filled with numerous organelles: mitochondria (*mit*), rough endoplasmic reticulum (*RER*), glycogen granules (*black arrowheads*). Nucleus (*N*), myofibrils (*mf*). Transverse, ultrathin section, TEM. *Scale bar*: 1 μm

The confocal analyses, performed using the Alexa-Phalloidin 546, revealed that in the studied species, the newly developed somite formed vesicles located on both sides of the neural tube (stage I) at the posterior (tail) part of the embryo. The wall of the vesicles was composed of tightly connected epithelial cells surrounding the somitocoel (Fig. 1a). Somitogenesis showed that in a more advanced development of the somites, the anterior-posterior gradient of muscle differentiation manifested in the anterior part of the embryo (sup Fig. 1). Therefore, in the anterior part of the embryo (stage II), the somites were already differentiated into the ventromedially located mesenchymal sclerotome, the laterally situated epithelial dermomyotome and the myotome between these (Fig. 1b). These observations were consistent with the anterior to posterior progression of somitogenesis typically seen in vertebrate embryos.

The dermomyotome was characterised by well-developed dorsomedial (DM) and ventrolateral (VL) lips (Fig. 1b). The TEM analysis revealed that the muscle progenitor cells located near to the dermomyotome closely adhered to each other. Their ultrastructure showed large nuclei with a narrow rim of cytoplasm, rich in mitochondria and rough endoplasmic reticulum (RER) and well-developed Golgi apparatus (AG) (Fig. 1b inset). During the subsequent developmental stages, some of the post-mitotic myotome cells became elongated. Also, some of the cells located in the myotome were characterised by proliferative activity, as was confirmed in the TEM (Fig. 1c) and with the confocal microscope for the immunocytochemical detection of phosphorylated histone H3 (stage III) (Fig. 1c inset) to visualise mitotic activity. At the same developmental stage, the elongated post-mitotic cells differentiated into mononucleated myotubes, which was confirmed by the presence of an incompletely developed contractile apparatus. Numerous organelles (such as RER, mitochondria and AG, surrounded by small vesicles and glycogen granules) were observed in the sarcoplasm of the mononucleated myotubes. It should be noted that the elongated nuclei of the myotubes had a homogeneous content (Fig. 1d). As myogenesis proceeded (stage IV), the dermomyotome cells were no longer observed (Fig. 1e). At the next developmental stage (stage V), for the first time in the myotomes, multinucleated myotubes occurred (Fig. 1f). The TEM analysis showed that the myotube sarcoplasm was rich in mitochondria, RER and glycogen granules. Furthermore, the myofibrils were regularly arranged, which is characteristic for a mature contractile apparatus (Fig. 1g).

### Unique features of myotomal myogenesis in the grass snake

**Fig. 2:**
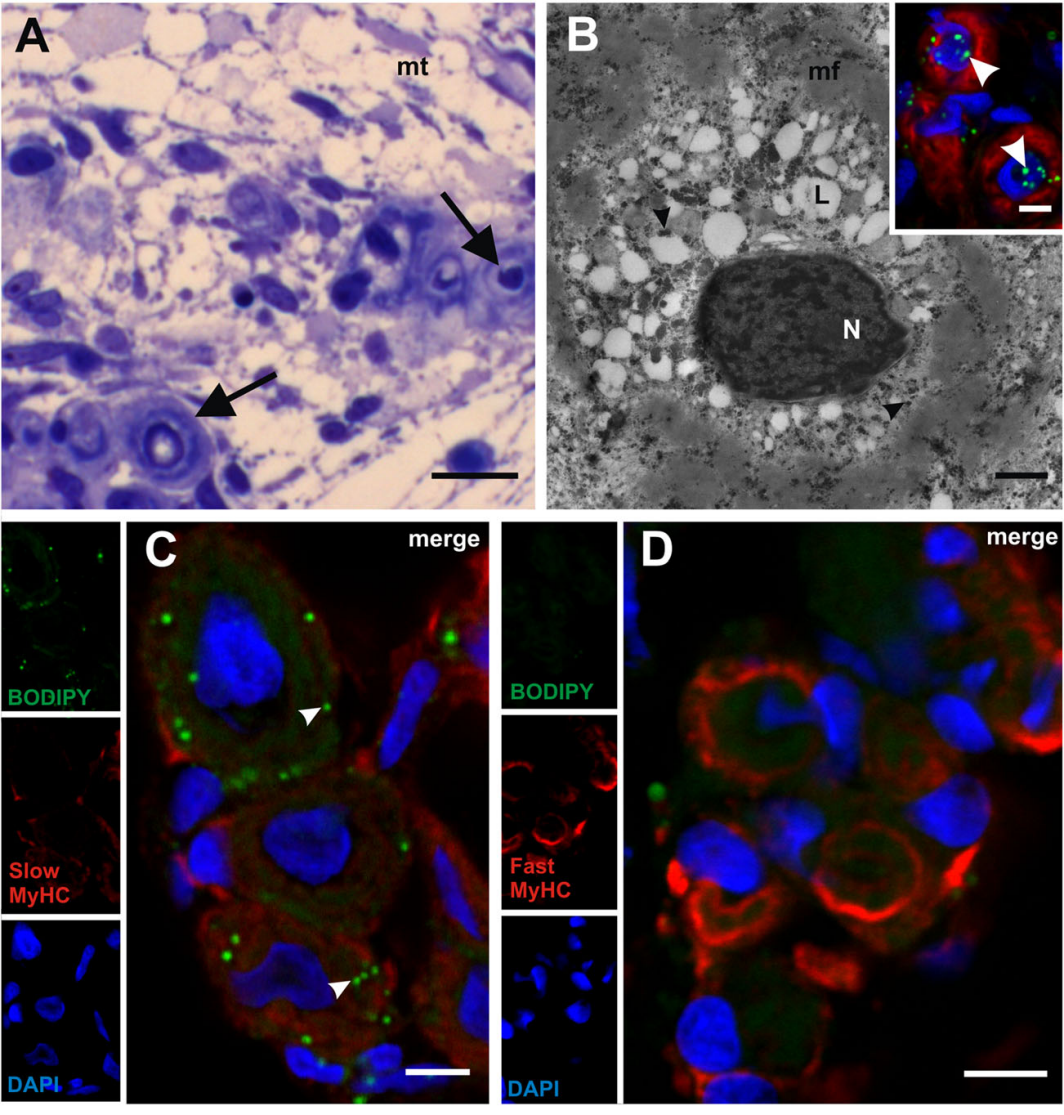
Unique features of myotomal myogenesis in grass snake. **a** Stage V. Structure of anterior myotome (*mt*) with the second class of myotubes (*black arrows*). Transverse, semithin section, methylene blue staining. *Scale bar*: 10 μm. **b** Stage V. Ultrastructure of second class of muscle fibre with centrally located heterogeneous nuclei (*N*) surrounded by numerous lipid droplets (*L*), myofibrils (*mf*) and glycogen granules (*black arrowheads*). Transverse, ultrathin section, TEM. *Scale bar*: 1 μm. *Inset*: detection of lipid droplets (*white arrowheads*). F-actin (*red*), nuclei (*blue*). Transverse, cryosection, confocal microscope. *Scale bar*: 5 μm. **c** Stage V. Detection of lipid droplets (*green*, *white arrowheads*) in slow muscles (*red*, anti-SlowMyHC antibody). Nuclei (*blue*). Transverse, cryosection, confocal microscope. *Scale bar*: 5 μm. **d** Stage VI. Absence of lipid droplets (*left*, *upper corner of figure*) in fast muscles (*red*, anti-FastMyHC antibody). Nuclei (*blue*). Transverse, cryosection, confocal microscope. *Scale bar*: 5 μm

Studies using the light microscope and the TEM revealed that two classes of myotubes developed during the grass snake myogenesis (stages V–VI). The first class was represented by typical muscle fibres with myofibrils that were located in the whole sarcoplasm. In the second class of muscle fibres, myofibrils were located in the peripheral sarcoplasm, and its central region was filled with lipid droplets, as confirmed by the histochemical staining with BODIPY (Fig. 2a, b). It is noteworthy that in the second class of muscle fibre sarcoplasm, the myofibrils did not form a regular arrangement. Furthermore, in the nucleus, numerous patches of heterochromatin were observed. Our studies showed that the lipid droplets were present only in those muscles which expressed a slow MyHC, whereas no lipid droplets were observed in the fast MyHC-positive fibres (Fig. 2c, d). These described features were not characteristic for the typical muscle fibres that appear during myogenesis in other vertebrates.

### Muscle growth

**Fig. 3:**
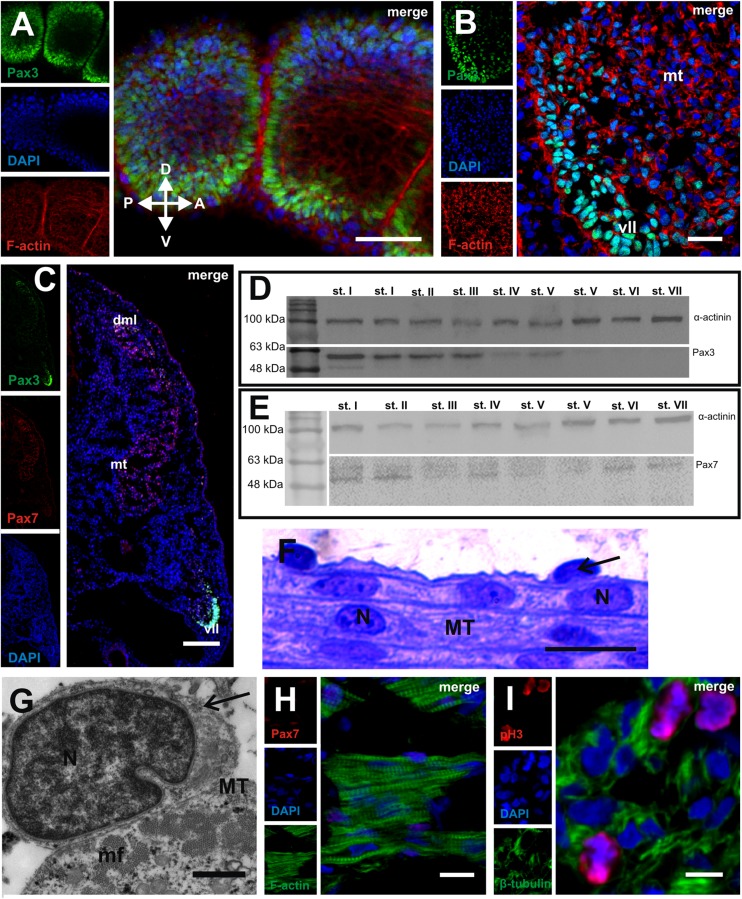
Muscle growth. **a** Stage I. Immunodetection of Pax3 protein (*green*) in posterior part of embryo. F-actin (*red*), nuclei (*blue*). Whole mount, confocal microscope. *Scale bar*: 50 μm. **b** Stage II. Immunodetection of Pax3 protein (*green*) in anterior part of embryo. F-actin (*red*), nuclei (*blue*), myotome (*mt*), ventrolateral lip of dermomyotome (*vll*). Transverse, cryosection, confocal microscope. *Scale bar*: 20 μm. **c** Stage II. Immunodetection of Pax3 protein (*green*) and Pax7 protein (*red*) in anterior part of embryo. Nuclei (*blue*), myotome (*mt*), derso-medial (*dml*) and ventro-lateral (*vll*) lips. Transverse, cryosection, confocal microscope. *Scale bar*: 50 μm. **d** Western blot analysis of Pax3 protein expression during successive developmental stages. Pax3 is marked together with an α-actinin band used as a loading control. The highest level of Pax3 protein in stage I, a steady decrease up to stages IV and V (7th day after oviposition) and its disappearance from stage V (ninth day after oviposition). **e** Western blot analysis of Pax7 protein expression during successive developmental stages. Pax7 is marked together with an α-actinin band used as a loading control. Approximately constant level in all studied developmental stages (I–VI). **f** Stage V. Structure of myotome filled with multinucleated myotubes (*MT*) with accompanying mononucleated cells (*black arrows*). Nuclei (*N*). Longitudinal, semithin section, methylene blue staining. *Scale bar*: 10 μm. **g** Stage V. Ultrastructure of mononucleated cell (*black arrow*) accompanying myotube (*MT*). Heterogeneous nuclei (*N*), myofibrils (*mf*). Transverse, ultrathin section, TEM. *Scale bar*: 1 μm. **h** Stage V. Immunodetection of Pax7 protein (*red*) in anterior myotome. Nuclei (*blue*), F-actin (*green*). Transverse, cryosection, confocal microscope. *Scale bar*: 10 μm. **i** Stage V. Immunodetection of phosphorylated histone H3 (*red*). Nuclei (*blue*), β-tubulin (*green*). Transverse, cryosection, confocal microscope. *Scale bar*: 5 μm

The immunocytochemical detection of the Pax3 protein (a marker of muscle progenitor cells that is upregulated during myogenesis) revealed that, in the newly formed somites, this protein was expressed in the somite wall cells (stage I) (Fig. 3a). As the somites became differentiated into three compartments (the dermomyotome, myotome and sclerotome), this protein was detected in both DM and VL lips of the dermomyotome and in the mononucleated cells of the myotome (stage II) (Fig. 3b, c). Also, at the studied developmental stage, the Pax7 protein (a marker of early proliferating myoblasts and progenitors of the satellite cells) was observed in the medial part of the dermomyotome and in some mononucleated cells of the myotome (Fig. 3c).

We also examined the expression levels of the Pax3/7 proteins during successive developmental stages, using the Western blot method. Our analysis of the Pax3 expression confirmed that the highest level of that protein occurred in stage I. We observed a slight decrease in the Pax3 level in later stages (II, III), and a further reduction of the signal occurred in stages IV and V (the 7th day after oviposition). The oldest analysed stages (V [the 9th day after oviposition], VI and VII) did not show detectable levels of the Pax3 protein (Fig. 3d). The immunoblot analysis of the Pax7 protein revealed its presence in all of the studied developmental stages (Fig. 3e).

The light and TEM studies revealed that the mononucleated cells closely adhered to the surface of the multinucleated myotubes (stage V). The ultrastructure of these cells showed a large nucleus that was rich in heterochromatin and with a narrow rim of cytoplasm (Fig. 3f, g). The presence of the Pax7 protein in the nucleus of these cells was detected immunocytochemically (Fig. 3h). Furthermore, these cells showed mitotic activity (Fig. 3i). Our observations, based on the light, TEM and the confocal microscopes, suggested that these cells were satellite cells involved in muscle growth due to their fusion with the myotubes.

## Discussion

### Somitogenesis, muscle differentiation and growth

A few studies of the muscle differentiation in this highly divergent group of animals have been carried out on the sand lizard (*Lacerta agilis*), the Egyptian cobra (*Naja haje*), the Chinese soft-shelled turtle (*Pelodiscus sinensis*) and the American alligator (*Alligator mississippiensis*), which represent various modes of locomotion (Nagashima et al. 2005; Rupik et al. 2012; Kusumi et al. 2013; Khannoon et al. 2016). Because the subject of reptilian trunk muscle development still remains elusive, we decided to shed more light on this topic. We carried out our investigation on the muscle differentiation and growth in the grass snake (*N. natrix*) by the use of light, TEM, and a confocal microscope and with Western blotting techniques. In the studied species, as in other vertebrates, the somitogenesis showed an anterior-posterior gradient (sup Fig. 1). The newly formed somites were composed of tightly connected epithelial cells surrounding the cavity called the somitocoel. Our studies revealed that the epithelial cells of the somite wall were Pax3-positive which is consistant with Pax 3 being a regulator of Myo D and Myf5 which are myogenic regulator factors responsible for initiation of muscle development and differentiation (see Parker et al. 2003). The arrangement of cells in the somite blocks (epithelial somite wall surrounding somitocoel; Fig. 1a) resembled the arrangement of cells described in birds and mammals (Takahashi and Sato 2008; reviewed by Bentzinger et al. 2012), and also in the sand lizard. These data in the grass snake, taken together with previous studies on other Amniota, suggest that in all Amniota, the pattern of somitogenesis is similar and significantly different from the process that is observed in anamniotes (fish and amphibians) (Daczewska and Kielbowna 2000; Daczewska 2001; Kacperczyk and Daczewska 2006, 2008; Kacperczyk et al. 2009, 2011; Rupik et al. 2012).

As myogenesis proceeds, the somite splits into three compartments: the epithelial dermomyotome, the myotome composed of mononucleated cells and the ventrolaterally located sclerotome. In the dermomyotome, two lips can be distinguished: dorsomedial (DM) and ventrolateral (VL). Similar structures have been observed during bird somite differentiation (Kahane et al. 1998; Kalcheim and Ben-Yair 2005). In the studied species, we also distinguished different populations of Pax3/Pax7-positive cells in the dermomyotome. Both the dorsomedial and ventrolateral lips contained the Pax3 protein, whereas a Pax7 expression was observed in the medial part of the dermomyotome. Moreover, Pax7-positive cells were detected in some mononucleated cells among the differentiating myotubes. The time course of the Pax3/Pax7 expression described here is similar to what has been shown to occur in birds (Galli et al. 2008). Both of the proteins were co-expressed during the early somitogenesis. In chick, a high Pax3 expression was observed in the DML and VLL, while Pax7 was synthesized in the central dermomyotome (Ben-Yair and Kalcheim 2005). Conversely, in mice, the Pax7 comes after the Pax3, and the early somites do not express Pax7 (Kassar-Duchossoy et al. 2005). Previous studies conducted on chick and mice have revealed that the fate of Pax3/Pax7 cells is different. Pax3-positive cells have been confirmed as muscle progenitor cells, which are capable of differentiating into muscle fibres (Kassar-Duchossoy et al. 2005; Relaix et al. 2005; Galli et al. 2008; reviewed by Buckingham and Relaix 2007). On the other hand, the expression of the Pax7 protein is characteristic for those muscle stem cells known as satellite cells (Gros et al. 2005; Relaix et al. 2005; Zammit 2006). Previous studies revealed that the Pax7 deficiency resulted in complete absence of satellite cells (Seale et al. 2000; Parker et al. 2003). It is worth noting that our analysis of the Pax3 protein expression during successive developmental stages showed a gradual decline, until its complete disappearance. In contrast, the Pax7 synthesis occurred at approximately the same level in all of the studied developmental stages. Similar observations have been made during observations of mouse and sand lizard myotomal muscle development (Horst et al. 2006; Rupik et al. 2012).

In the grass snake myotome, the progenitors of the muscle fibres started to elongate and differentiate into mononucleated myotubes with an incompletely developed contractile apparatus. At the next developmental stage, multinucleated myotubes appeared for the first time in the myotomes, accompanied by mononucleated cells. Their location, ultrastructure and their Pax7 protein expression resembled those of satellite cells (as reviewed by Bentzinger et al. 2012; Yin et al. 2013). Thus, we are convinced that the cells described above are involved in muscle growth.

### Unique features of grass snake myogenesis

During our investigation, we observed two classes of muscle fibres. The first class was represented by typical muscle fibres, with myofibrils located throughout the sarcoplasm. In the second class of muscle fibres, myofibrils in an irregular arrangement were located in the peripheral sarcoplasm, and the central region was filled with lipid droplets. Furthermore, during our studies, we confirmed that lipid droplets were only present in the slow muscle fibres, whereas no lipid droplets were observed in the fast muscles. The above-described features, confirmed by our immunocytochemical studies, are not characteristic for the typical muscle fibres that appear during myogenesis in other vertebrates. This observation is in agreement with the results obtained from a study of *Naja haje* myogenesis (Khannoon et al. 2016) which indicated that the muscles capable of storing lipid droplets were slow muscles. According to these authors, lipid droplets are the most economical form of storing energy and are used during hibernation. We therefore suggest that the fat-rich muscles appearing during snake myogenesis could be treated as an energy source during hibernation even though this is not a common feature in reptiles. Furthermore, our unpublished data revealed an absence of lipid droplets during sand lizard (*Lacerta agilis*) muscle differentiation. As such, we can hypothesise that lipid droplet storage during the trunk muscle myogenesis is a snake-specific feature. Further investigations based on physiological and biochemical methods are necessary to fully explain this phenomenon.

Our studies of muscle growth and differentiation in the grass snake revealed similarities to the amniote model of myogenesis. However, we also observed differences in the advanced stages of muscle development. We believe that the unique features of snake myogenesis that have been described may depend on environmental conditions and the habitats occupied by this group of vertebrates. In conclusion, we also suggest that the model of myotomal myogenesis in reptiles, birds and mammals shows the consistent morphological and molecular features. We strongly believe that the grass snake, in spite of the unique features of its myogenesis, fits into the amniote-specific model of trunk muscle development.

## Electronic supplementary material

Below is the link to the electronic supplementary material.

Supplementary Figure 1 Anterior-posterior gradient of somitogenesis and muscle differentiation. **a** Stage II. Structure of anterior part of embryo. Immunodetection of myosin heavy chains (green) in the anterior myotome (mt). F-actin (red), NT – neural tube, mt – myotome. Transverse, cryosection, confocal microscope, Scale bar: 10 μm. **b** Stage II. Structure of medial part of embryo. Immunodetection of myosin heavy chains (green) in the medial myotome (mt). F-actin (red), NT – neural tube, mt – myotome. Transverse, cryosection, confocal microscope, Scale bar: 10 μm. **c** Stage II. Structure of posterior somites (white arrows). F-actin (red), anterior (A), posterior (P), dorsal (D), ventral (V). Whole mount staining, confocal microscope. Scale bar: 5 μm. **d** Stage II. Structure of two terminal somites (white arrows). F-actin (red), anterior (A), posterior (P), dorsal (D), ventral (V). Whole mount staining, confocal microscope. Scale bar: 5 μm. (GIF 599 kb)

High resolution image (TIF 25477 kb)
