# Supplementary figures and images for: Does the grass snake (Natrix natrix) (Squamata: Serpentes: Natricinae) fit the amniotes-specific model of myogenesis?
Source: Protoplasma. 2016 Nov 10;254(4):1507–16. doi: 10.1007/s00709-016-1040-5 (PMC5487930; doi:10.1007/s00709-016-1040-5)

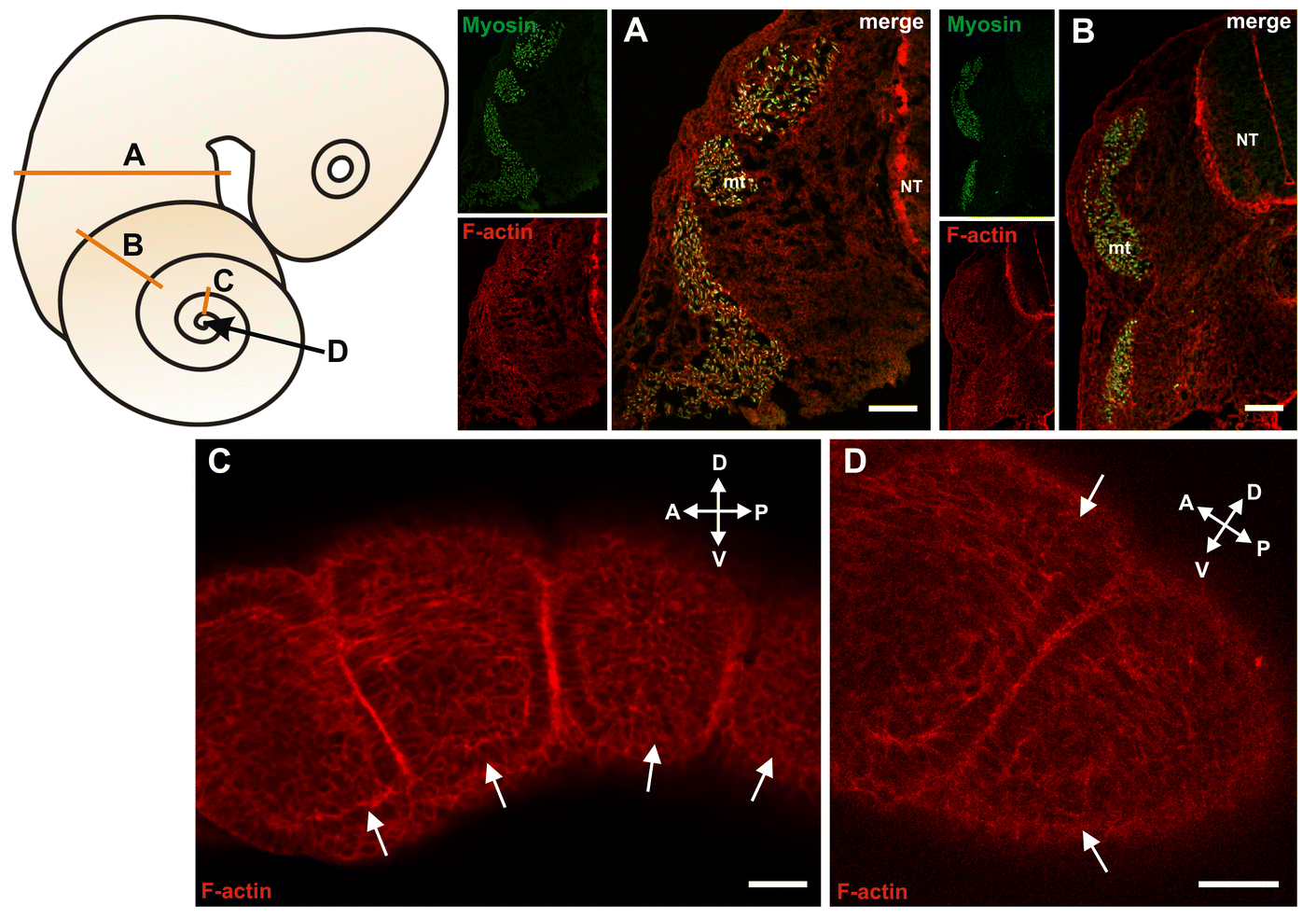

Supplement: Supplementary file 1 — Anterior-posterior gradient of somitogenesis and muscle differentiation. a Stage II. Structure of anterior part of embryo. Immunodetection of myosin heavy chains (green) in the anterior myotome (mt). F-actin (red), NT – neural tube, mt – myotome. Transverse, cryosection, confocal microscope, Scale bar: 10 μm. b Stage II. Structure of medial part of embryo. Immunodetection of myosin heavy chains (green) in the medial myotome (mt). F-actin (red), NT – neural tube, mt – myotome. Transverse, cryosection, confocal microscope, Scale bar: 10 μm. c Stage II. Structure of posterior somites (white arrows). F-actin (red), anterior (A), posterior (P), dorsal (D), ventral (V). Whole mount staining, confocal microscope. Scale bar: 5 μm. d Stage II. Structure of two terminal somites (white arrows). F-actin (red), anterior (A), posterior (P), dorsal (D), ventral (V). Whole mount staining, confocal microscope. Scale bar: 5 μm. (GIF 599 kb) [file 709_2016_1040_Fig4_ESM.gif]

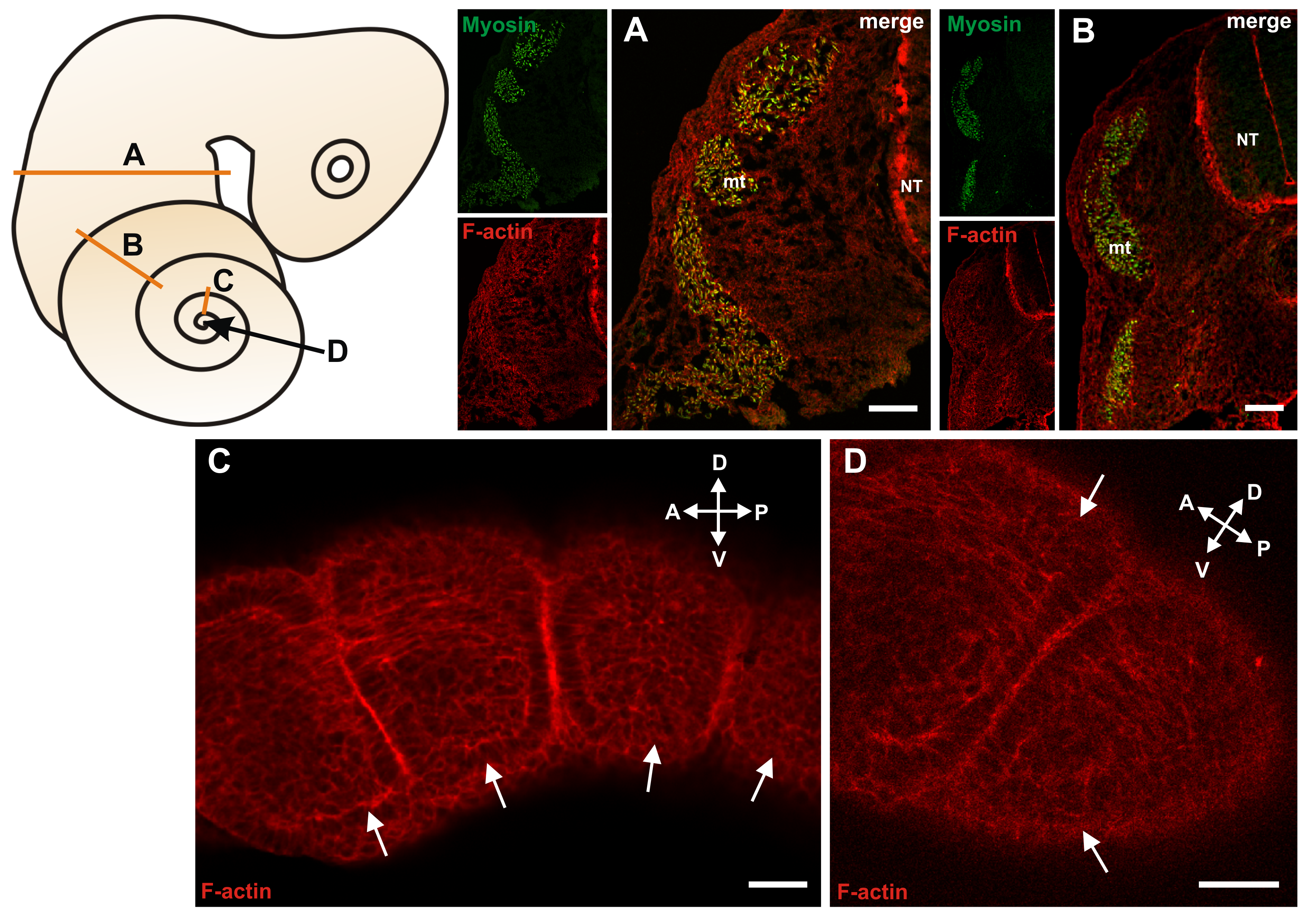

Supplement: Supplementary file 2 — High resolution image (TIF 25477 kb) [file 709_2016_1040_MOESM1_ESM.tif]
